# Assessing the efficacy of large language models in spinal health information dissemination

**DOI:** 10.1016/j.bas.2024.102815

**Published:** 2024-04-12

**Authors:** Aaron Lawson McLean, Christian Senft

**Affiliations:** Department of Neurosurgery, Jena University Hospital – Friedrich Schiller University Jena, Jena, Germany

**Keywords:** Large language models, Healthcare AI, Patient education, Spinal health, Regulatory frameworks, Ethical implications

## Abstract

• Examines AI's role in spinal health education.
• Stresses multidisciplinary AI evaluations.
• Advocates for stringent AI healthcare regulations.

The manuscript by Lang et al. titled "Are large language models valid tools for patient information on lumbar disc herniation? A spine surgeons' perspective” and published in the current issue of the journal, probes the effectiveness of large language models (LLMs) in conveying patient education on lumbar disc herniation, shedding light on the broader conversation surrounding the intersection of artificial intelligence (AI) and healthcare delivery (Lang et al., 2024). This inquiry is critical amid the rapid integration of generative AI technologies into patient education, underscored by the imperative for these technologies to produce outputs that are not only accurate and reliable but also comprehensible to the general public. The study focuses on evaluating LLMs – such as ChatGPT and Google Bard (now Gemini) – from the perspective of spine surgeons, analyzing the models' capacity for clarity, empathy, and accuracy in communication. While revealing insights into the potential role of LLMs in patient education, the research also exposes a complex web of methodological and conceptual challenges that invite a more detailed and nuanced conversation about AI's role in healthcare.

At the heart of this discourse lies the methodological approach adopted by Lang et al. which, while pioneering, underscores the inherent challenges in evaluating AI-driven tools within the medical field. The reliance on a homogenous group of spine surgeons as the sole evaluators introduces a layer of subjectivity and potentially narrows the scope of the study's applicability. This methodological design speaks to a broader issue within the field of healthcare AI research: the critical importance of multidisciplinary evaluations in assessing AI tools. The complex nature of healthcare, with its myriad stakeholders including patients, healthcare professionals, ethicists, and regulators, necessitates a comprehensive evaluative framework that encompasses diverse perspectives. Such an approach would not only enhance the validity and generalizability of research findings but also ensure that AI applications are aligned with the multifaceted needs and expectations of the healthcare ecosystem.

Moreover, the findings reported by Lang et al. characterized by a variability in evaluations and the presence of unsatisfactory responses, open a window into the ongoing debate over AI's reliability and accuracy in healthcare contexts. This variability is not merely a reflection of the subjective nature of human evaluations but also highlights the intrinsic limitations of current AI models. Despite significant advancements in AI technologies, the capacity of LLMs to understand and replicate the nuances of medical knowledge and patient communication remains imperfect. This observation is a microcosm of the broader challenges facing the integration of AI in healthcare: balancing the potential of AI to revolutionize patient education and care with the imperative for these technologies to be rigorously validated for clinical accuracy and safety.

The manuscript's discussion of regulatory considerations, notably the impending EU AI Act, serves as a segue into one of the most pressing issues in the field of healthcare AI: the development and implementation of regulatory frameworks that are capable of keeping pace with the rapid evolution of AI technologies. The regulation of AI in healthcare presents a complex puzzle, involving not only the technical aspects of AI performance and safety but also ethical considerations surrounding patient autonomy, privacy, and equity (Meskó and Topol, 2023). As AI models become increasingly integrated into healthcare delivery and patient education, the need for robust, dynamic regulatory frameworks that can adapt to technological advancements while safeguarding patient interests has never been more critical. This regulatory challenge is compounded by the global nature of AI development and deployment, calling for international collaboration in setting standards and guidelines that ensure the safe, ethical, and effective use of AI in healthcare.

Furthermore, the integration of AI into patient education, as explored by Lang et al. prompts a broader reflection on the future trajectory of AI in healthcare. The potential of AI to democratize access to medical knowledge, personalize patient education, and enhance the patient-care provider relationship is immense (Mittlelstadt, 2021). Yet, realizing this potential hinges not only on overcoming technical and regulatory hurdles but also on addressing fundamental ethical questions. How can AI be leveraged to enhance patient autonomy without supplanting the human element of healthcare? In what ways might AI exacerbate or mitigate existing disparities in healthcare access and outcomes? These questions underscore the need for a forward-looking approach to AI integration that is rooted in ethical principles, prioritizes patient-centered outcomes, and embraces the complexities of healthcare delivery.

In conclusion, the manuscript by Lang et al. represents a significant contribution to the burgeoning field of healthcare AI, providing valuable insights into the use of LLMs in patient education. However, the study also serves as a catalyst for a broader, more critical discourse on the future of AI in healthcare. As the field progresses, it is imperative that research methodologies evolve to reflect the multidisciplinary nature of healthcare, that regulatory frameworks adapt to the dynamic landscape of AI technologies, and that ethical considerations remain at the forefront of AI integration efforts. Only through a holistic, collaborative approach can the promise of AI in healthcare be fully realized, enhancing patient education, care, and outcomes in an era of technological transformation.

## Declaration of competing interest

The authors declare that they have no known competing financial interests or personal relationships that could have appeared to influence the work reported in this paper.
